# From digital chest tomosynthesis to 3D CT

**DOI:** 10.1093/rpd/ncaf162

**Published:** 2026-03-13

**Authors:** Attila Simkó, Patrik Sund, Maral Mirzai, Jonas Ivarsson, Åse Johnsson, Angelica Svalkvist, Magnus Båth

**Affiliations:** Department of Biomedical Engineering and Medical Physics, Sahlgrenska University Hospital, Region Västra Götaland, SE-413 45, Gothenburg, Sweden; Department of Biomedical Engineering and Medical Physics, Sahlgrenska University Hospital, Region Västra Götaland, SE-413 45, Gothenburg, Sweden; Department of Medical Radiation Sciences, Institute of Clinical Sciences, Sahgrenska Academy, University of Gothenburg, SE-413 45, Gothenburg, Sweden; Department of Biomedical Engineering and Medical Physics, Sahlgrenska University Hospital, Region Västra Götaland, SE-413 45, Gothenburg, Sweden; Department of Medical Radiation Sciences, Institute of Clinical Sciences, Sahgrenska Academy, University of Gothenburg, SE-413 45, Gothenburg, Sweden; Department of Applied IT, University of Gothenburg, SE-412 96, Gothenburg, Sweden; Department of Radiology, Institute of Clinical Sciences, Sahlgrenska Academy, University of Gothenburg, SE-413 45, Gothenburg, Sweden; Department of Radiology, Sahlgrenska University Hospital, Region Västra Götaland, SE-413 45, Gothenburg, Sweden; Department of Biomedical Engineering and Medical Physics, Sahlgrenska University Hospital, Region Västra Götaland, SE-413 45, Gothenburg, Sweden; Department of Medical Radiation Sciences, Institute of Clinical Sciences, Sahgrenska Academy, University of Gothenburg, SE-413 45, Gothenburg, Sweden; Department of Biomedical Engineering and Medical Physics, Sahlgrenska University Hospital, Region Västra Götaland, SE-413 45, Gothenburg, Sweden; Department of Medical Radiation Sciences, Institute of Clinical Sciences, Sahgrenska Academy, University of Gothenburg, SE-413 45, Gothenburg, Sweden

## Abstract

Digital chest tomosynthesis refers to the 3D reconstruction of low-dose projection images acquired within a limited angular range. The reconstructions have lower depth resolution and are more prone to motion artifacts compared to computed tomography (CT). While recent deep learning approaches aim to reconstruct full-resolution CT volumes from projections, they are computationally demanding due to the high resolution and inherently 3D nature of the task. In this study, we propose a more efficient alternative. Our deep learning-based framework reconstructs sagittal CT slices from small patches of projection data, significantly lowering memory demands. Rather than predicting continuous Houndsfield unit (HU) values, we segment voxels into air, soft tissue, or bone classes. Our results show that the method captures coarse structural features and depth information with high consistency, but struggles to reconstruct fine details. While not yet suitable for clinical deployment, the approach highlights a promising direction for low-resource tomosynthesis-based volumetric imaging.

## Introduction

Chest X-ray remains one of the most commonly performed imaging studies in radiology due to its wide availability, low cost, and relatively low radiation dose. However, its two-dimensional nature limits the ability to accurately localize small or subtle features. Computed tomography (CT) significantly improves upon conventional chest X-ray by providing high-resolution, 3D imaging [1]. Despite its diagnostic advantages, the relatively high radiation dose associated with CT restricts its routine or frequent use especially in screening or follow-up settings. Digital chest tomosynthesis has emerged as a promising low-dose alternative, offering pseudo-volumetric imaging through the reconstruction of limited-angle X-ray projections [2]. While it improves lesion detectability compared to standard radiography, it remains limited by reduced depth resolution and susceptibility to motion artifacts, which can hinder diagnostic accuracy in some clinical scenarios.

To address the limitations of digital chest tomosynthesis in clinical imaging, we propose a deep learning-based framework to synthesize full-resolution CT-like volumes directly from projection images acquired during the tomosynthesis imaging. Although deep learning has been previously applied to tomosynthesis reconstruction [3], recent trends such as Neural Radiance Fields (NeRFs) [4, 5] and other 3D generative methods [3, 6] are often unnecessarily complex, we argue. These models require large amounts of memory, extended training time, and access to expensive, high-end graphics processing units (GPUs)—factors that limit their practical adoption. A scoping review [7] urges the field to explore new deep learning architectures pointing out that deep learning has already shown promising results in the field. Instead of increasing architectural complexity, we demonstrate that careful design choices can significantly simplify the reconstruction problem.

A major challenge in generating CT-like volumes from tomosynthesis projections is the reliance on supervised learning, which requires paired tomosynthesis and CT data. Such datasets are difficult to obtain due to inter-modality variation and the difficulty of achieving accurate registration. We overcome this issue by creating synthetic projection images from the CT volumes, directly ensuring alignment [3]. This strategy eliminates the need for aligned data, and enables more scalable training, as corresponding projection images are no longer necessary, and they can be generated from any CT volume. However, for models trained on synthetic data, it must be ensured that it also generalizes well to real data.

While recent advances in deep learning have leaned heavily toward deeper architectures and more elaborate 3D representations, such complexity often comes at the cost of interpretability, efficiency, and accessibility. We argue that increasing model depth or dimensionality is not always the most effective path forward—especially in clinical settings where computational resources may be limited and real-time performance is critical. Instead, our proposed method focuses on simplifying the reconstruction task through informed design decisions that align more closely with the structure of the data and the physical properties of the task to solve. We propose reframing the problem and reducing unnecessary complexity, and hence taking the first step toward generating high-quality volumetric images from projection images.

## Approach

In the following section we describe our dataset, and our contributions. Our proposed method is built around two key simplifications. First, we use a sagittal slice-wise reconstruction approach that predicts a CT slice from corresponding 2D patches of projections images, significantly reducing computational complexity. We use sagittal slices because the vertical tube sweep naturally aligns with this orientation, simplifying the geometric correspondence between projections and target slices. Second, instead of regressing continuous Hounsfield Unit values, the model can alternatively classify each voxel into coarse tissue categories—air, soft tissue, or bone—transforming the task into a segmentation problem. This categorical output can serve as a lightweight surrogate for full CT synthesis in applications where relative tissue differentiation is sufficient. Together, these design choices constitute a novel, efficient, and flexible framework for volumetric imaging from low-dose image inputs.

### Dataset

Data were collected from 2967 patients within the scope of the Swedish CArdioPulmonary bioImage Study [8] (ethical review ref. 2021–03857). For each patient, raw projection data, reconstructed image series, and volumetric CT images were available. All CT scans were acquired on a SOMATOM Definition Flash scanner (Siemens Healthcare, Forchheim, Germany) at 120 kV using automatic tube current modulation (CareDose4D, quality reference 25 mAs) a pitch of 0.6. Images were reconstructed on a 512 × 512 in-plane matrix with ~512 axial slices, using I50f kernel with a slice thickness of 0.75 mm and an increment of 0.6 mm.

The tomosynthesis examinations were performed using the GE Definium 8000 system with the VolumeRAD option (GE Healthcare, Chicago, Illinois, United States). For all examinations, 60 low-dose images (2048 × 2048 voxels) were collected in a vertical linear sweep of the X-ray tube.

As the patient positioning is significantly different between the two scans, they cannot be directly used for supervised learning.

### Synthetic projection generation

**Figure 1:**
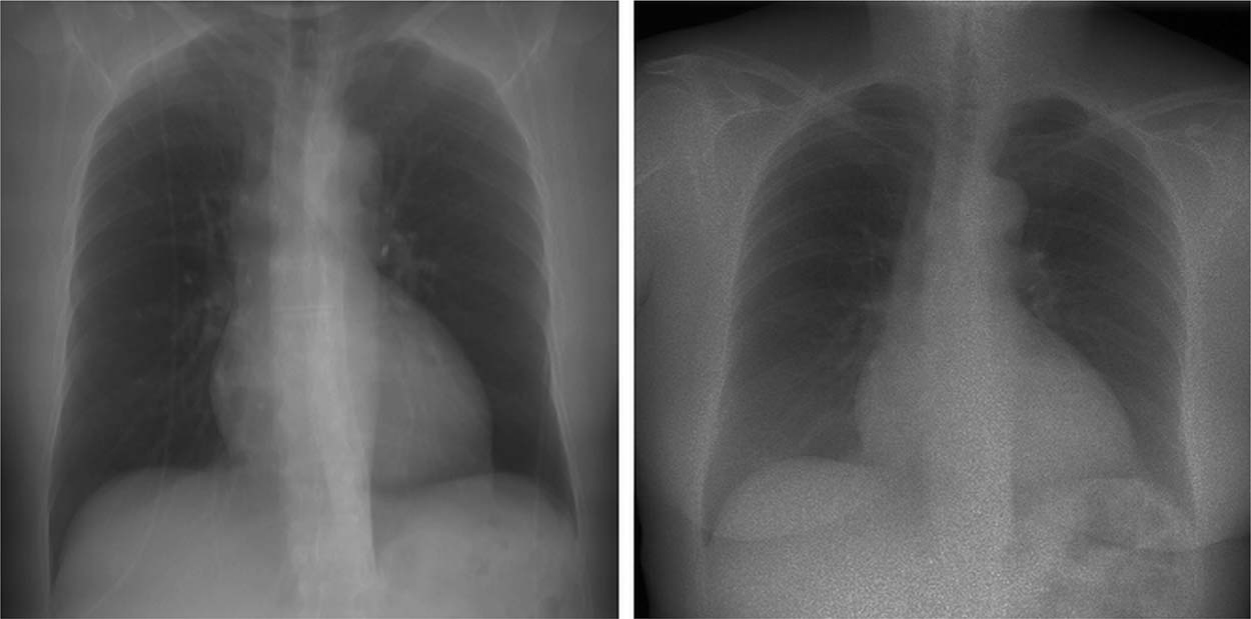
A real projection image (left) and the synthetic projection image generated from the corresponding CT volume (right) showing anatomical misalignment between acquisitions.

The geometry of acquiring the projection images is well-defined and known. The X-ray tube is moving vertically from −15° to 15°, with a horizontal distance of 180 cm between the detector plane and the source. The same geometry was implemented in ASTRA Toolbox [9, 10], an open-source GPU-accelerated library for tomographic projection and reconstruction operations. CT volumes were converted to electron density maps using standard HU-to-density conversion tables. Forward projections were then computed using ray-tracing through the volume, followed by physics-based augmentation including beam hardening, scatter, motion blur, and Poisson noise. Figure 1 shows an example of the real and generated projections for the same patient. This ensures that the patient positioning is the same for both projection and CT data. As the model learns a geometric mapping between the input projections and the 3D space, patient alignment will not influence the generalization of the model.

### Slice-wise approach

**Figure 2:**
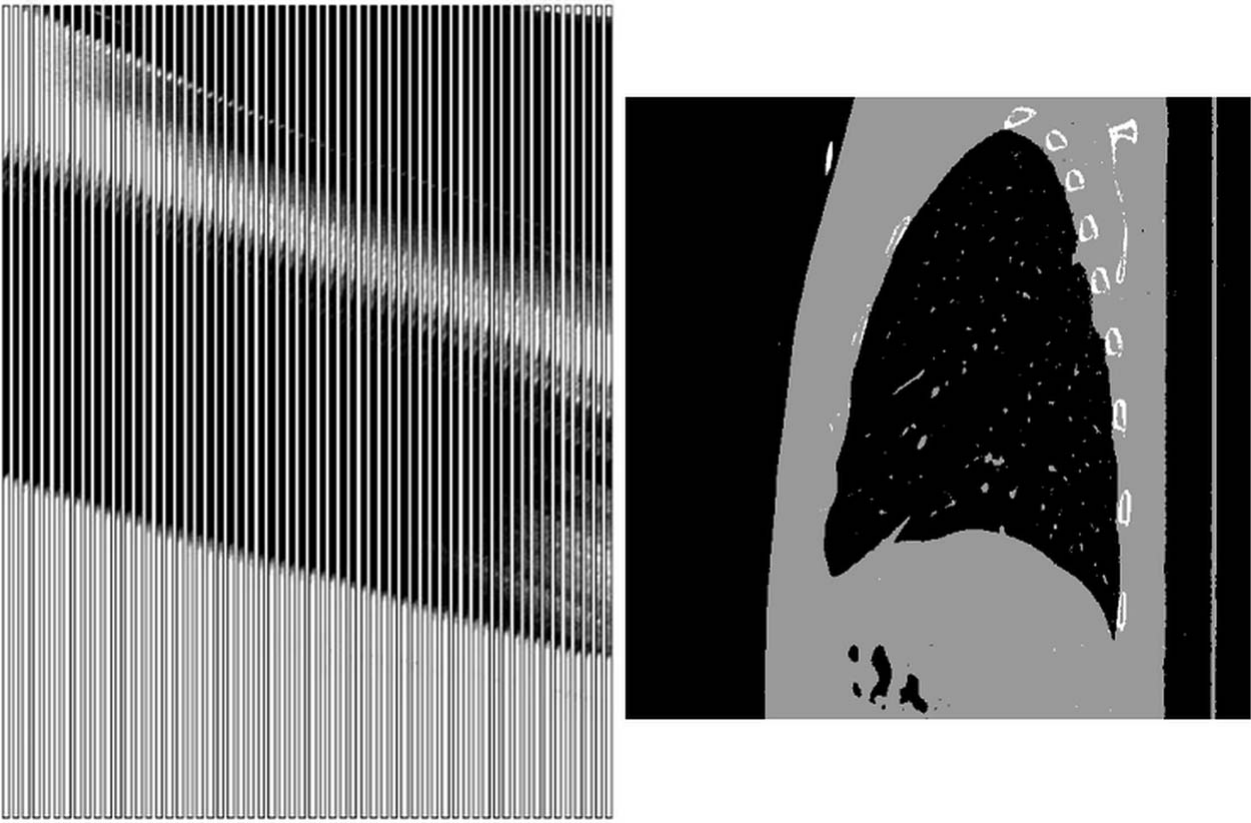
An example input (60 projection patches, left) output (corresponding sagittal CT slice, right) pair. Due to the generation approach, they can now be used for supervised training.

Instead of using the entire 2048 × 2048 projection images to predict the full 512 × 512 × 512 CT volume, we simplify the task by leveraging knowledge about the problem. During acquisition of the projection images, the X-ray source moves along a vertical line. While reconstructing the complete 3D volume typically requires all projection data, reconstructing a single sagittal slice only depends on a localized region within each projection image. Due to the geometry of the scan and magnification effects, a narrow vertical strip around the sagittal plane contains all relevant information. As a result we extract a smaller patch—specifically, 31 pixels wide—from each projection and use it as an input to predict the corresponding sagittal slice. An example input–output pair is visualized in Fig. 2. This significantly reduces the input size while still preserving all necessary information for accurate reconstruction of the sagittal slice.

### Segmentation alternative

The main goal is the detailed reconstruction of fine structures, such as lung nodules, and their shapes, hence, the actual HU values of the reconstructions are less important. We begin by converting the CT volume into a segmentation map of air (below –800HU), soft tissue (above –800HU and below 300HU) and bone (above 300HU). This turns our task into a segmentation problem. This classification approach is simpler than continuous HU regression and focuses the model on capturing structural boundaries. For visualization and evaluations, the segmentations are later converted to CT-like images by assigning –1000HU for air, 0HU for soft tissue, and 500HU for bone.

### Model architecture

Our model architecture is a hybrid 2.5D U-Net (an encoder-decoder architecture using skip-connections to preserve spatial detail) designed to efficiently map limited-angle tomosynthesis projection data to high-resolution CT-like outputs. The input is a stacked 3D volume of 60 projection patches (60 × 2048 × 31). The encoder processes this 3D input using a series of convolutional blocks to extract both local and contextual features. The encoder extracts 3D features, which are decoded into more memory-efficient 2D sagittal slices. We use adaptive skip connections to preserve spatial detail. The final output applies softmax activation to each pixel independently, producing class probabilities that sum to one for air, soft tissue, and bone.

### Training process

The dataset was divided into training (2467 patients), validation (250 patients), and test (250 patients) subsets. The model was trained using the Adam optimizer [11] with an initial learning rate of 3 × 10^−4^, and a composite loss function combining generalized Dice and categorical cross-entropy to address class imbalance and promote segmentation accuracy. Training used a batch size of 16 on a single NVIDIA T40 GPU (24 GB), requiring 400 epochs and ~190 h to converge. The inference time per patient is ~21 s.

## Results

The proposed method was evaluated using both quantitative metrics and qualitative, visual assessments of the effectiveness of CT reconstruction from the projections. Evaluations were conducted on the synthetic test sets as well as real-world tomosynthesis data to assess generalization performance.

### Quantitative evaluation

We evaluated the model using both segmentation-based metrics and intensity based error measures to capture different aspects of reconstruction performance. Since the output is categorical, with predictions for air, soft tissue, and bone classes, we computed a confusion matrix to assess class-wise agreement between the predicted segmentation and ground truth CT-derived labels which is collected in Table 1.

**Table 1:** Confusion matrix showing row-normalized voxel classification results based on combined voxels for all patients. Rows correspond to *true classes*, and columns to *predicted classes*.

		Predicted values		
		Air (<–800HU)	Soft tissue	Bone (>300HU)
True values	Air	0.9355	0.0614	0.0031
	Soft tissue	0.1011	0.8331	0.0658
	Bone	0.2516	0.3677	0.3806

In addition to segmentation metrics, we also computed the mean absolute error (MAE) between predicted and reference HU values, using the converted class labels to representative HU values to estimate the difference in intensity. The mean absolute error (MAE) and its standard deviation was 91.2 ± 34.6 HU overall, with 527.4 ± 78.9 HU for bone, 104.2 ± 8.0 HU for soft tissue, and 69.1 ± 9.0 HU for air. As expected, bone regions showed higher MAE due to their sharp gradients and higher susceptibility to artifacts in limited-angle projection data. Notably, the MAE values are not directly comparable across classes, due to the discretized output space; however, they still reflect relative trends in reconstruction quality.

### Qualitative evaluation

**Figure 3:**
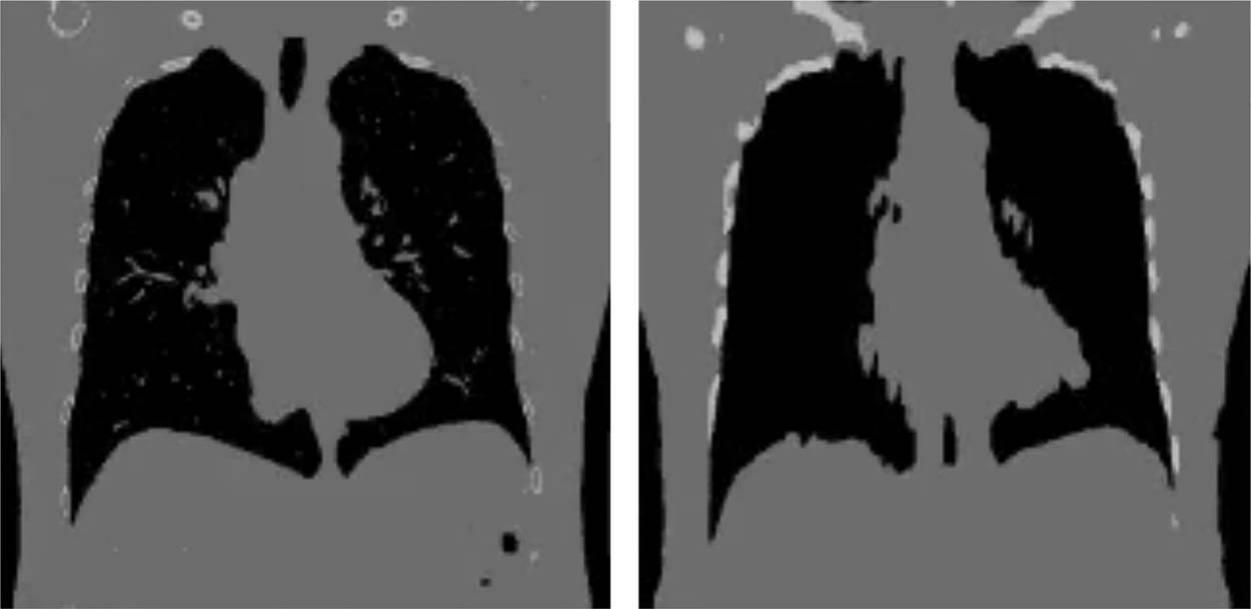
A ground truth coronal slice (left) and the corresponding model prediction (right) reconstructed from output sagittal slices using our proposed model.

The model performance was further examined qualitatively to assess anatomical consistency between the slices and clinical realism. Figure 3 shows representative coronal slices predicted by the model (sagittal slice-by-slice) compared to ground-truth CT, highlighting the structural fidelity of major anatomical regions.

**Figure 4:**
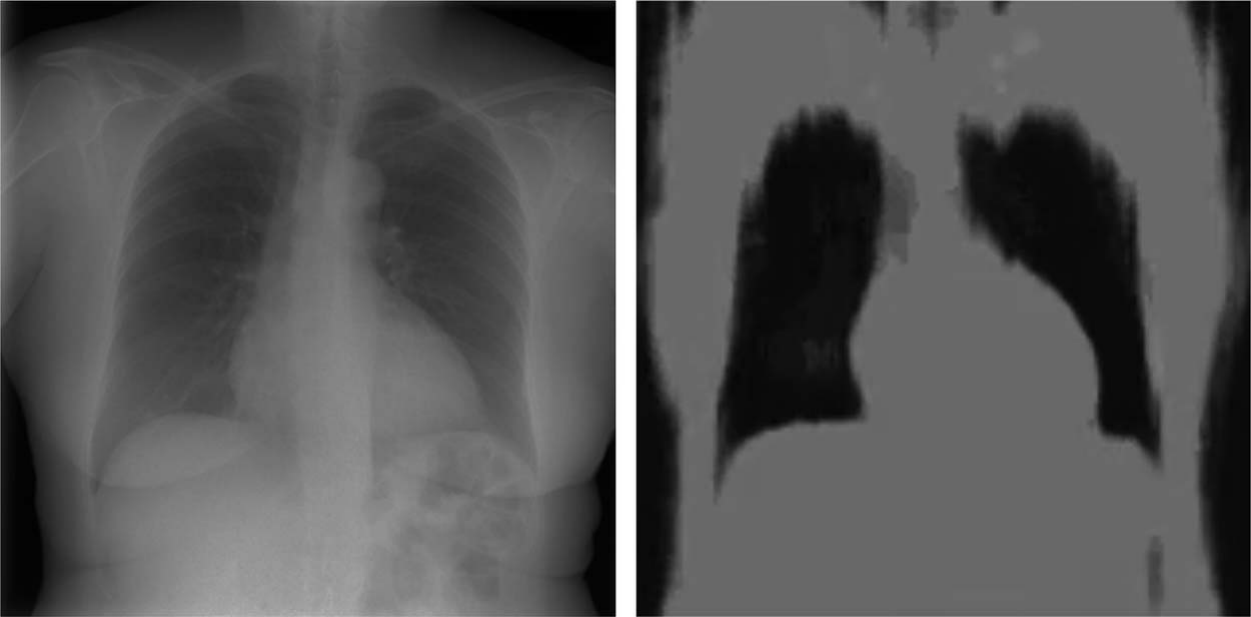
Real projection image (left) and corresponding coronal reconstruction from our proposed model predictions (right) showing limited structural detail.

To evaluate generalizability, the model also predicted CT volumes from real tomosynthesis projection data. An example is visualized in Fig. 4.

## Discussion

Our self-supervised approach eliminates the dependency on precisely registered projection-CT datasets, making it more practical for real-world applications. By enabling CT-quality volumetric imaging from low-dose tomosynthesis projections, this approach could support dose reduction in screening programs and improve access to 3D imaging in resource-limited settings. The use of synthetic projection images ensures robust training while mitigating registration errors.

Our model uses only 1.3 million parameters—substantially fewer than recent volumetric methods like ρ-NeRF and MedNeRF, which each require ~14 million. This efficiency is made possible by reformulating the reconstruction task as a 2D problem, predicting sagittal slices independently rather than synthesizing full 3D volumes. While this limits volumetric context, it significantly reduces memory and computational requirements, making the approach more practical for deployment in resource-constrained settings.

The confusion matrix reveals strong overall classification performance, particularly for air and soft tissue, which show high true positive rates and minimal misclassification. However, bone tissue remains the most challenging class, with a substantial proportion of voxels misclassified as soft tissue. This reflects the inherent difficulty in segmenting bone structures from limited-angle projections due to their high density, overlapping anatomy, and sparse representation in the training data. The performance imbalance across classes highlights the need for loss functions that account for such skew, which motivated our use of the generalized Dice loss. By weighting classes inversely to their frequency, this loss helped mitigate the dominance of more prevalent classes and encouraged the model to better learn underrepresented structures like bone.

Further quantitative evaluation showed good overall segmentation performance, with low MAE for air and soft tissue, and higher error in bone due to sharp intensity transitions. However, as the qualitative results illustrate, the model struggles to reconstruct fine details such as small nodules—an issue not fully captured by the quantitative metrics.

Despite using a slice-wise approach, the model demonstrates strong consistency across the volume, with smooth transitions and no abrupt discontinuities between the slices. However, more in-depth qualitative evaluation highlights significant limitations of the model. As shown in Fig. 3 even on synthetic data, the predicted reconstruction lacks fine anatomical detail and appears overly smooth, failing to capture smaller internal structures present in the ground truth. In Fig. 4 the output on real tomosynthesis data is heavily blurred and largely devoid of meaningful internal anatomy, indicating poor generalization. However, the model still manages to capture coarse anatomical layout, suggesting that the underlying approach has potential, and could benefit from improved supervision, architectural refinement, or domain adaptation to better preserve fine structures and generalize to clinical data.

While continuous HU regression offers greater fidelity, our results suggest that many structural and lesion-relevant features can be effectively inferred using lightweight, patch-based 2D models that classifies into coarse tissue types approximating HU values. This challenges the assumption that 3D modeling is necessary for synthetic CT generation from chest tomosynthesis.

Future work will focus on incorporating real tomosynthesis data to improve model generalization, exploring hybrid 2.5D–3D architectures to enhance depth perception, implementing more classification categories for soft tissue types, integrating an adversarial loss term for self-supervised learning, and evaluating clinical relevance through dedicated reader studies.

## Conclusion

We present a deep learning framework for reconstructing full-resolution CT volumes from projection data using a self-supervised strategy. By leveraging synthetic projections and a 2.5D U-Net model, we achieve effective CT synthesis while managing memory limitations. While our results show promising spatial coherence and memory efficiency, there are clear limitations regarding the reconstruction of anatomical details, highlighting opportunities for improvements.
